# Sports Activity after Low-contact-stress Total Knee Arthroplasty – A long term follow-up study

**DOI:** 10.1038/srep24630

**Published:** 2016-04-19

**Authors:** Ines Vielgut, Lukas Leitner, Norbert Kastner, Roman Radl, Andreas Leithner, Patrick Sadoghi

**Affiliations:** 1 Department of Orthopaedic Surgery, Medical University of Graz, Austria

## Abstract

The purpose of this study was to provide comprehensive long-term data about sports activity levels in patients following total knee arthroplasty (TKA) and to determine the impact of pre-operative function, pain and specific performed sports on the results. 236 patients who have undergone TKA for severe osteoarthritis of the knee were asked to provide specific information regarding exercised types of sports before surgery and after at least 10 years following TKA. Pre- and postoperative function and pain were evaluated by the use of Tegner-, WOMAC- and VAS Score. After a mean of 14.9 years, a significant improvement regarding pain and function was observed. Pre-operative Tegner- and WOMAC scores revealed significant positive correlations with the post-operative Tegner-Score. Accordingly, a high percentage of patients (70.9%) stayed actively involved in sports. Nevertheless, the number of performing patients has decreased according to the sports impact. 71.3% continued practising low-impact-, 43.7% intermediate-impact sports whereas only 16.4% kept performing high impact sports. We conclude that TKA is highly effective in long-time pain reduction as well as improvement of function. Additionally, we found considerable sports activities preserved in the investigated series. However, sports activities in particular, seem to decrease according to the impact of sports.

Osteoarthritis of the knee joint is defined by the progressive loss of articular cartilage associated with synovial inflammation and structural changes of the subchondral bone. It is a highly common musculoskeletal disease, accompanied by pain and functional limitations and therefore often responsible for a considerable loss of patients’ quality of life^1^. The understanding of the impact of both biological and psychosocial factors on the development of the disease should provide the basis for individual treatment strategies^2^. These strategies include non-invasive treatment options such as physical therapy and behaviour therapeutic measures, supported by adequate pain management, if necessary. Non-pharmacologic behavioural treatment, recommended for both therapy and prevention of osteoarthritis, may include lifestyle adjustment strategies to change unhealthy nutritional- and physical activity habits. Physiotherapy includes isometric muscle activation and strengthening as well as exercises to increase the aerobic capacity. This option is most suitable for patients with mild osteoarthritis who pursue a still sporty lifestyle^3^. Surgical treatment strategies for advanced osteoarthritis of the knee joint include a partial or total knee replacement: These procedures are considered to be a successful treatment for end-stage osteoarthritis of the knee regarding pain relief as well as functional improvement. Total knee arthroplasty (TKA) was performed predominantly in the elderly, already retired patients for a long time in the past^4^.

In recent years, a steady rise of patients undergoing TKA could be observed, probably due to higher functional demands in order to provide an active lifestyle despite of advanced arthritis of the knee or age^5^,^6^. These circumstances have led to an increased number of performed TKAs in both young, still working patients as well as in patients of all ages with high sportive requirements^7^,^8^,^9^.

The purpose of this follow-up study was to provide comprehensive long-term data about sports activity levels in patients following TKA and to determine whether pre-operative function, pain and specific performed sports activities do influence the outcome.

## Methods

This study followed accepted ethical, scientific and medical standards and was conducted in compliance with recognized international standards, including the principles of the Declaration of Helsinki. Informed consent was obtained from all study participants. The study protocol was approved by the Ethics Committee of the Medical University of Graz, Austria (23-284 ex 10/11).

### Patients

Our arthroplasty database identified 236 patients (260 knees) who have undergone total knee arthroplasty between 1986 and 2002 and have been actively engaged in sports prior to surgery. All patients received a cemented total knee arthroplasty with rotating platform (LCS) without patellar-resurfacing at the same institution.

Eight patients were lost to follow-up and therefore excluded. The data of the remaining patients (236) was available for final analysis. The patients consisted of 193 female and 43 male patients with an average age of 62.7 ± 11.4 years.

121 patients received TKA on the right, 91 on the left side. 24 patients underwent bilateral total knee arthroplasty.

### Indication, surgical technique and postoperative complications

The indication for TKA was either primary or secondary grade IV osteoarthritis of the knee. All pre-operative radiographs were assessed using the Kellgren and Lawrence classification system^10^.

All arthroplasties were performed through a medial para-patellar approach at the same department. A cemented total knee arthroplasty (LCS^®^ COMPLETE Mobile Bearing Total Knee System, DePuy Synthes, Warsaw, IN) with a rotating-platform polyethylene was used for all patients. Postoperative complications were observed in seven patients: Aseptic implant loosening occurred in three patients, implant failure (implant breaking) in one, polyethylene-wear in one and early periprosthetic infection in one patient as well. One patient suffered from postoperative arthrofibrosis. The mean follow-up time was 14.9 ± 3.0 years. The demographic data is illustrated in Table 1.

### Assessment of sports activity

Included patients were asked to provide specific information regarding exercised types of sports before surgery and after a minimum of 10 years following TKA. The indicated sports were categorized according to their impact in low-, intermediate and high-impact sports. High impact sports included jogging, handball, volleyball, basketball, soccer, and squash, intermediate impact sports were such as badminton, inline-skating, tennis, downhill skiing, cross-country skiing, riding, bowling and rock climbing and low impact sports included cycling, hiking, Nordic-walking, gymnastics, fitness/weight lifting, dancing, swimming and golf.

### Assessment of function, activity levels and pain

Pre- and postoperative function and pain were evaluated the by the use of the Tegner Acitivity level Scale^11^, the Visual Analogue Scale (VAS)^12^ and the Western Ontario & McMaster Universities Osteoarthritis Index (WOMAC)^13^. All of these evaluation scores were obtained immediate preoperatively and during the final follow up (14.9 ± 3.0 years).

The Tegner activity level scale is a graduated list of activities of daily living, recreation, and competitive sports. The patients were asked to select the level of participation that best describes their current level of activity before surgery and 10 years after TKA. The WOMAC consists of a set of standardized questionnaires to evaluate the condition of patients with osteoarthritis of the knee including pain, stiffness, and function of the joint.

The Index is graded on a scale from 0 (asymptomatic) to 96 (worst score). Tegner Scale and VAS range from 0 to 10.

### Statistical analysis

Due to the retrospective design of the study, we have calculated “Observed Post Hoc Power” according to Hoenig and Heisey^14^. According to this method, post hoc power for differences with a p-value < 0.01 reveals a value greater than 80% of power.

Spearman correlation coefficient was used to calculate the strength of the correlation between two variables. Comparison of metric variables between two groups was performed using t-test if normally distributed. In all calculations a two-sided p-value < 0.05 was considered to be statistically significant. SPSS software version 17.0 for Windows (SPSS, Chicago, IL) was used for data analysis.

## Results

### Function and activity levels

**Figure 1:**
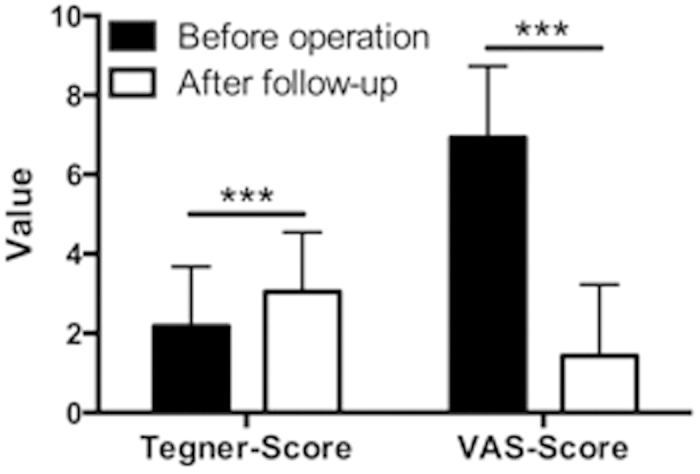
Tegner and VAS-Score of 236 patients evaluated preoperatively and at minimum follow-up of ten years - (14.9 ± 3.0 years) after implantation of the low-contact-stress total knee arthroplasty.

In order to provide a standardized method of grading work and sporting activities, the Tegner Acitivity Level Scale was obtained immediate preoperatively and after a mean follow-up time of 14.9 years. According to that, we observed a significant improvement of the preoperative status (Tegner 3.04 ± 1.5 at final follow-up; pre-operatively 2.18 ± 1.5) (Fig. 1).

Furthermore, the pre-operative Tegner- and WOMAC Scores revealed significant positive correlations with the post-operative Tegner Score (r = 0.38, p < 0.001 and r = 0.65, p < 0.001). These results can be interpreted in the sense that preoperative preserved function and sports activities do influence the functional outcome positively.

Age was a significant negative predictor for both the pre- and post-operative Tegner-Score (r = −0.21, p = 0.03 and r = −0.31, p < 0.001).

Post-surgery complications, as referred to above, were significantly negative correlated with the postoperative Tegner-Score (3.71 ± 1.0; pre-operatively 3.19 ± 1.3). No significant differences were detected between women and men in terms of pre-and postoperative obtained evaluation scores.

### Pain

After a mean follow-up time of 14.9 years, a significant improvement of the pre-operative status in terms of pain VAS 1.42 ± 1.8 at final follow-up; pre-operatively 6.93 ± 1.8 could be observed as well.

These results are shown graphically in Fig. 1.

### Specific sports activities

Before undergoing TKA, all of the patients included in this study practised at least one of the mentioned sports regularly. After a mean follow-up of 10 years following TKA 169 (70.8%) patients stayed actively involved in sports.

71.3% of the patients continued practising low-impact sports, especially sports as Swimming, Hiking, Nordic Walking, Gymnastics and Fitness Training were continued to be exercised.

43.7% continued with intermediate-impact sports, especially Badminton and Inline Skating. Sports like Tennis and Skiing on the other hand have lost all its active participants over the years.

The worst result could be observed for high impact sports. 83.3% were unable to continue high-impact sports, such as Jogging, Soccer, Handball, Volleyball etc. following TKA.

Detailed specification on performed sports activities preoperatively and at follow-up is presented in Table 2.

According to the magnitude of differences between per- and postoperative scores, we observed a post hoc power greater than 80% for the WOMAC (p < 0.001) and Tegner Score (p < 0.001) according to Hoenig and Heisey^14^.

## Discussion

The purpose of this study was to provide comprehensive data about sports activity levels and the long-term outcome regarding function and pain following TKA of the knee.

Although several patients do return to high-level sports following TKA, it is not unhesitatingly recommended by performing surgeons or other occupational groups involved in the patients’ treatment^15^. This position is supported by studies that have shown a higher risk for early implant failure such as aseptic loosening and increased wear caused by greater activity^16^,^17^,^18^,^19^,^20^. Other studies, by contrast, have found no host factors, particularly increased activity, associated with loosening and implant wear and TKA is suggested to be an effective and safe treatment for younger patients as well^21^,^22^,^23^. But even the supporters of sport activities following TKA suggest predominantly sports with minimal impact to the knee, for example swimming, cycling, or power walking^24^. The 1999 Knee Society Survey developed a consensus recommendation for sports participation following TKA. They have classified aerobics (low impact), bicycling (stationary), walking, bowling, golf, Ballroom-dancing, swimming, or horseback-riding, amongst others, as harmless for patients following TKA. Activities not recommended include for example soccer, tennis, volleyball, handball, football, basketball, and jogging^25^,^26^.

However, the actual activity is individual and determined by several factors, at least the personal responsibility of each patient and his attending surgeon.

In our series we had some patients who continued practising high-impact sports such as jogging, handball, volleyball, soccer, and squash following TKA. However, it must be stated that the percentage was very low, in particular when compared to the preoperative data.

According to the Tegner Score, we observed a significant improvement of functional activity in patients who have undergone TKA for severe osteoarthritis of the knee. This finding is supported by a recent study of Stone *et al*., who described a significant correlation between Kellgren and Lawrence classification and functional improvement in patients who have undergone TKA for severe osteoarthritis, especially for grade III and IV^27^.

However, sports activity in particular, has decreased according to the impact of sports, from high-, intermediate- to low-impact, in the investigated series.

A closer look into the patient demographics permits conclusions regarding the age as a limiting factor in terms of sports activity, especially when considering our long follow-up period. Age, in fact, was recorded to have a negative impact on both pre-and postoperative activity levels. Accordingly, it can be assumed that particularly elder patients do not increase their activity levels following TKA, despite of the significant reduction of pain, which could be observed for the majority of the patients.

Although TKA is considered to be a highly successful treatment option in terms of pain reduction for patients suffering from end-stage osteoarthritis, the results can be manifold. It is known that about 20% of patients continue complaining about persisting pain of the affected knee^28^,^29^,^30^. There may be a great range of causes, ranging from aseptic loosening, instability, anterior knee pain due to patellar maltracking or malbalanced TKAs, over psychological factors such as depression or anxiety to patient related factors that may influence the outcome negatively such as age, gender or comorbidities^31^,^32^,^33^,^34^. In our series we observed a significant improvement of pain despite of the rather long follow-up period.

We hereby want to underline the following limitations of our work: The information provided in this manuscript may be used to inform patients about the expected long-term results in terms of pain-relief, functional improvement, and ability to perform sports after TKA. Nonetheless, this is a retrospective analysis of prospective collected data. A further limitation is certainly the lack of a matched control group. In order to attain a higher level of evidence, a prospective study should be conducted, which is naturally difficult for arthroplasty studies with a follow-up of ten years. However, we want to underline the benefit that our study evaluated a group of patients with a minimum follow-up oft ten years, operated on and evaluated by standardized methods.

## Conclusion

Although long-term pain reduction and improved function can be generally expected following TKA, we found evidence, that post-operative sports ability is positively correlated with pre-operative activity. In fact, we found considerable sports activities preserved in our population of patients, who have been actively involved to sports prior to surgery. Notwithstanding these benefits it must be stated that the ability to perform sports has decreased following TKA according to the sports impact. However, it must be pointed out that a large part of the patients was able to continue doing low-impact sports.

Moreover, with regard to our results related to the functional outcome following TKA, it may be concluded that, independent of the extent of degeneration of the knee joint due to osteoarthritis, the functional outcome is depending to a significant degree on the preoperative functional status as well. We therefore recommend our patients to preserve or improve their functional status in terms of joint mobility and active participation of adapted sports for as long as possible before undergoing TKA in order to achieve the best possible results. Possible strategies may include intensive preoperative movement- and physiotherapy.

## Additional Information

**How to cite this article**: Vielgut, I. *et al*. Sports Activity after Low-contact-stress Total Knee Arthroplasty – A long term follow-up study. *Sci. Rep*. **6**, 24630; doi: 10.1038/srep24630 (2016).

## Figures and Tables

**Table 1:** Demographic data of 236 patients (260 knees) who underwent TKA and were followed-up for a minimum time of ten years (14.9 ± 3.0 years).

Patient demographics	n
Female	193
Male	43
Bilateral	24
Right knee	121
Left knee	91
Excluded	8
Total	236

**Table 2:** Evaluation of sports activities of 236 patients after implantation of a low-contact-stress (LCS) total knee arthroplasty.

Activity	Preoperative (%)	Postoperative (%)	Relative Change
Low Impact			
Cycling	44.2	26.1	−18.1
Hiking	43.3	26.4	−16.9
Nordic Walking	30	21.7	−8.3
Gymnastics	28.9	21.7	−7.2
Fitness/Weight Training	3.3	3.3	0
Dancing	18	8.7	−9.3
Swimming	26.7	19.6	−1.7
Golf	2.2	2.2	0
Intermediate Impact			
Badminton	7.8	3.3	−4.5
Inline Skating	6.7	1.1	−5.6
Tennis	4.4	0	−4-4
Downhill Skiing	10	0	−10
Cross-country skiing	5.6	0	−5.6
Riding	1.1	0	−1.1
Martial Arts	0	0	0
Bowling	3.4	3.3	−0.1
Rock Climbing	0	0	0
High Impact			
Jogging	12.2	2.2	−10
Handball	4.4	1.1	−3.3
Volleyball	7.8	1.1	−6.7
Basketball	5.6	0	−5.6
Soccer	11.1	2.2	−8.9
Squash	6.7	1.1	−5.6

## References

[b1] Castrogiovanni P. & Musumeci G. Which is the Best Physical Treatment for Osteoarthritis? J. Funct. Morphol. Kinesiol. 1, 54–68 (2016).

[b2] Pereira D., Ramos E. & Branco J. Osteoarthritis. Acta Med. Port. 28, 99–106 (2015). 25817486 10.20344/amp.5477

[b3] Musumeci G. . Advantages of exercise in rehabilitation, treatment and prevention of altered morphological features in knee osteoarthritis. A narrative review. Histol Histopathol 29(6), 707–719 (2014). 24452819 10.14670/HH-29.707

[b4] Lombardi A. V. . Do Patients Return to Work After Total Knee Arthroplasty? Clin Orthop Relat Res. 472(1), 138–46 (2014). 23761175 10.1007/s11999-013-3099-z PMC3889457

[b5] Carr A. J. . Knee replacement. Lancet. 379, 1331–1340 (2010). 22398175 10.1016/S0140-6736(11)60752-6

[b6] Kastner N. . Impact of the tibial slope on range of motion after low-contact-stress, mobile-bearing, total knee arthroplasty. Int Orthop. 38(2), 291–295 (2014). 24346515 10.1007/s00264-013-2242-5 PMC3923942

[b7] Mancuso C. A., Ranawat C. S., Esdaile J. M., Johanson N. A. & Charlson M. E. Indications for total hip and total knee arthroplasties. Results of orthopaedic surveys. J Arthroplasty. 11, 34–46 (1996). 8676117 10.1016/s0883-5403(96)80159-8

[b8] Ravi B. . The changing demographics of total joint arthroplasty recipients in the United States and Ontario from 2001 to 2007. Best Pract Res Clin Rheumatol 26, 637–647 (2012). 10.1016/j.berh.2012.07.014 23218428

[b9] Abe H. . Jogging after total hip arthroplasty. Am J Sports Med. 42(1), 131–137 (2014). 24114754 10.1177/0363546513506866

[b10] Kellgren J. H. & Lawrence J. S. Radiological assessment of osteo-arthrosis. Ann. Rheum. Dis. 16(4), 494–502 (1957). 13498604 10.1136/ard.16.4.494 PMC1006995

[b11] Tegner Y. & Lysolm J. Rating Systems in the Evaluation of Knee Ligament Injuries. Clin Orthop Relat Res. 198, 43–49 (1985). 4028566

[b12] Huskisson E. C. Measurement of pain. Lancet. II, 1127–1131 (1974). 4139420 10.1016/s0140-6736(74)90884-8

[b13] Bellamy N., Buchanan W. W., Goldsmith C. H., Campbell J. & Stitt L. W. Validation study of WOMAC: a health status instrument for measuring clinically important patient relevant outcomes to antirheumatic drug therapy in patients with osteoarthritis of the hip or knee. J Rheumatol. 15(12), 1833–1840 (1988). 3068365

[b14] Hoenig J. M. & Heisey D. M. The abuse of power: The pervasive fallacy of power calculations in data analysis. Amer. Statist. 55, 19–24 (2001).

[b15] Schmidutz F. . Sports activity after short-stem hip arthroplasty. Am J Sports Med. 40(2), 425–32 (2012). 21993977 10.1177/0363546511424386

[b16] Odland A. N., Callaghan J. J., Liu S. S. & Wells C. W. Wear and lysis is the problem in modular TKA in the young OA patient at 10 years. Clin Orthop Relat Res. 469(1), 41–47 (2011). 20568028 10.1007/s11999-010-1429-y PMC3008910

[b17] Mayr H. O., Reinhold M., Bernstein A., Suedkamp N. P. & Stoehr A. Sports activity following total knee arthroplasty in patients older than 60 years. J Arthroplasty. 30(1), 46–49 (2011). 25304937 10.1016/j.arth.2014.08.021

[b18] Atwood S. A., Currier J. H., Mayor M. B. . Clinical wear measurement on low contact stress rotating platform knee bearings. J Arthroplasty. 23(3), 431 (2008). 18358384 10.1016/j.arth.2007.06.005

[b19] Harris W. H. Wear and periprosthetic osteolysis: the problem. Clin Orthop Relat Res. 393, 66 (2001). 11764372 10.1097/00003086-200112000-00007

[b20] Cherian J. J., Jauregui J. J., Banerjee S., Pierce T. & Mont M. A. What Host Factors Affect Aseptic Loosening After THA and TKA? Clin Orthop Relat Res. Feb 26 (2015). 10.1007/s11999-015-4220-2 PMC4488212 25716213

[b21] Bisschop R., Brouwer R. W. & Van Raay J. J. Total knee arthroplasty in younger patients: a 13-year follow-up study. Orthopedics. 33(12), 876 (2010). 21162506 10.3928/01477447-20101021-13

[b22] Mont M. A., Marker D. R., Seyler T. M. . High-impact sports after total knee arthroplasty. J Arthroplasty. 23(3), 80 (2008). 18722307 10.1016/j.arth.2008.04.018

[b23] Meier W. . Total knee arthroplasty: muscle impairments, functional limitations, and recommended rehabilitation approaches. J Orthop Sports Phys Ther. 38(5), 246–56 (2008). 18448878 10.2519/jospt.2008.2715

[b24] Healy W. L., Iorio R. & Lemos M. J. Athletic activity after total knee arthroplasty. Clin Orthop Relat Res. 65–71 (2000). 11064974 10.1097/00003086-200011000-00009

[b25] Hirschmann M., Testa E., Amsler F. & Friederich N. F. The unhappy total knee arthroplasty (TKA) patient: higher WOMAC and lower KSS in depressed patients prior and after TKA. Knee Surg Sports Traumatol Arthrosc. 21, 2405–2411 (2000). 23358576 10.1007/s00167-013-2409-z

[b26] Elson D. W. & Brenkel I. J. Predicting pain after total knee arthroplasty. J Arthroplasty. 21, 1047–1053 (2000). 17027550 10.1016/j.arth.2005.12.010

[b27] Stone O. D., Duckworth A. D., Curran D. P., Ballantyne J. A. & Brenkel I. J. Severe arthritis predicts greater improvements in function following total knee arthroplasty. Knee Surg Sports Traumatol Arthrosc. (2015) Oct 6. [Epub ahead of print]. 10.1007/s00167-015-3806-2 26441252

[b28] Hirschmann M. T. . A novel standardized algorithm for evaluating patients with painful total knee arthroplasty using combined single photon emission tomography and conventional computerized tomography. Knee Surg Sports Traumatol Arthrosc. 18(7), 939–944 (2010). 20148324 10.1007/s00167-010-1070-z

[b29] Wylde V., Hewlett S., Learmonth I. D. & Dieppe P. Persistent pain after joint replacement: prevalence, sensory qualities, and postoperative determinants. Pain. 152(3), 566–572 (2012). 21239114 10.1016/j.pain.2010.11.023

[b30] Toms A. D., Mandalia V., Haigh R. & Hopwood B. The management of patients with painful total knee replacement. J Bone Joint Surg Br. 91(2), 143–150 (2009). 19190044 10.1302/0301-620X.91B2.20995

[b31] Brander V., Gondek S., Martin E. & Stulberg S. D. Pain and depression influence outcome 5 years after knee replacement surgery. Clin Orthop Relat Res. 464, 21–26 (2007). 17603386 10.1097/BLO.0b013e318126c032

[b32] Brander V. A. . Predicting total knee replacement pain: a prospective, observational study. Clin Orthop Relat Res. 416, 27–36 (2003). 14646737 10.1097/01.blo.0000092983.12414.e9

[b33] Lingard E. A. & Riddle D. L. Impact of psychological distress on pain and function following knee arthroplasty. J Bone Joint Surg Am. 89(6), 1161–1169 (2007). 17545417 10.2106/JBJS.F.00914

[b34] Toms A. D., Mandalia V., Haigh R. & Hopwood B. The management of patients with painful total knee replacement. J Bone Joint Surg Br. 91(2), 143–150 (2009). 19190044 10.1302/0301-620X.91B2.20995

